# Immune-related gene signature predicts overall survival of gastric cancer patients with varying microsatellite instability status

**DOI:** 10.18632/aging.202271

**Published:** 2020-12-09

**Authors:** Ruyue Tian, Jiexuan Hu, Xiao Ma, Lei Liang, Shuilong Guo

**Affiliations:** 1 Department of Oncology, Beijing Friendship Hospital, Capital Medical University, Beijing 100050, China; 2 Department of Ultrasound, Aero Space Central Hospital, Beijing 100050, China; 3 Department of Gastroenterology, Beijing Friendship Hospital, Capital Medical University, National Clinical Research Center for Digestive Disease, Beijing Digestive Disease Center, Beijing Key Laboratory for Precancerous Lesion of Digestive Disease, Beijing 100050, China

**Keywords:** gastric cancer, microsatellite instability, immune-related genes, survival analysis, The Cancer Genome Atlas

## Abstract

Purpose: Gastric cancer (GC) is one of the most common and fatal malignancies globally. While microsatellite instability (MSI) index has earlier been correlated with survival outcome in gastric cancer patients, the present study aims to construct a risk-stratification model based on immune-related genes in GC patients with varying MSI status.

Results: The univariate and multivariate Cox regression analyses identified SEMA7A, NUDT6, SCGB3A1, NPR3, PTH1R, and SHC4 as signature genes, which were used to build the prognostic model for GC patients with microsatellite instability-low (MSI-L) and microsatellite stable (MSS). Whereas, for GC patients with microsatellite instability-high (MSI-H), prognostic model was established with three genes (SEMA6A, LTBP1, and BACH2), based on the univariate and multivariate Cox regression, and Kaplan-Meier survival analyses.

Conclusion: The prognostic immune-related gene signature identified in this study may offer new targets for personalized treatment and immunotherapy for GC patients with MSI-H or MSI-L/MSS status.

Methods: The Cancer Genome Atlas (TCGA) and ImmPort databases were used to extract expression data and to explore prognostic genes from the immune-related genes (IRGs), respectively. Univariate and multivariate Cox regression analysis were applied to identify IRGs correlated with patient prognosis. The regulatory network between prognostic IRGs and TFs were performed using R software.

## INTRODUCTION

Gastric cancer (GC) is the fifth most common malignancy and the third leading cause of cancer-related deaths worldwide [1]. Based on The Cancer Genome Atlas (TCGA) molecular characterizations, GC can be categorized into four subtypes: Epstein-Barr virus (EBV, 9%), microsatellite instability-high (MSI-H, 22%), genomically stable (GS, 20%), and chromosomal instability (CIN, 50%) [2]. These subtypes are strongly associated with patients' survival outcome [3].

Microsatellites, short tandem DNA sequence repeats, are widely distributed throughout the genome. Due to de-regulation in the DNA mismatch-repair (MMR) genes and proteins, these tandem repeats are prone to insertion-deletion events, causing microsatellite instability (MSI). Clinically, a tumor harboring two or more altered dinucleotide repeats in the microsatellite region is termed as MSI-H, while in other cases it is either termed as microsatellite instability-low or microsatellite stable (MSI-L/MSS) [4, 5].

Using MSI as a predictive value, especially in the early stage GC, patients with MSI-H status showed better survival outcomes compared to those with MSS [6, 7]. Moreover, therapeutically, patients with MSI-H tumors may benefit with PD-1 pathway blockade, and the MSI status also has been considered by the FDA as an indication for pembrolizumab treatments [8]. Despite these encouraging results, a significant proportion of MSI-H GC patients remain insensitive to immunotherapy [9], which necessitates the identification of molecular biomarkers for the prognostic screening of GC patients with MSI-H status.

Here, using TCGA datasets, we performed a differential gene expression analysis using IRGs in GC patients with MSI-H or MSI-L/MSS status. The selected genes were further analyzed using univariate and multivariate Cox regression analyses to establish their correlation with disease prognosis. Furthermore, we rigorously explored the expression status and prognostic landscape of IRGs and constructed an individualized risk-stratification model for MSI-H or MSI-L/MSS patients, respectively. And, we also explored the underlying transcriptional regulation of IRGs and their utility as prognostic signatures. Taken together, the risk-stratification model proposed in our study offers a foundation for subsequent immune-related work, which nevertheless requires further evaluation in clinics before consideration for prognosis of patients with gastric cancer.

## RESULTS

### Identification of differentially expressed immune-related genes (IRGs) in MSI-H samples of gastric cancer (GC)

**Figure 1:**
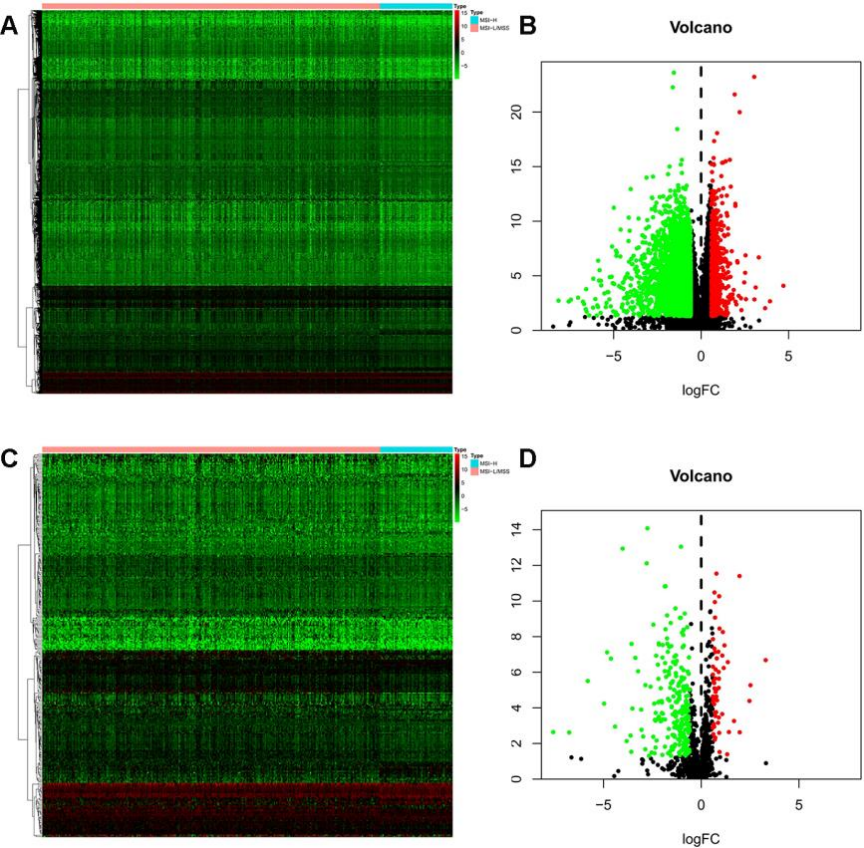
**Differentially expressed immune-related genes (IRGs).** (**A**, **B**) Heatmap and volcano plot of the upregulated (red) and downregulated (green) genes between the MSI-L/MSS and MSI-H samples of gastric cancer. (**C**, **D**) Profiling data of the IRGs are shown in heatmap and volcano plot, red dots represent upregulated and green dots represent downregulated. (MSI-H, microsatellite instability-high status; MSI-L, microsatellite instability-low status; MSS, microsatellite stable).

Gene expression data analysis of the 374 GC patient samples with variable MSI status allowed identification of 5268 differentially expressed genes (DEGs) which met the statistical threshold criteria. Amongst these, 540 genes were up-regulated while 4728 genes were significantly down-regulated in the MSI-H patient samples as compared to the MSI-L/MSS.(Figure 1A, 1B) And of these, 289 DEGs were screened as immune-related genes. (Figure 1C, 1D).

### Prognostic value of genes in gastric cancer

**Figure 2:**
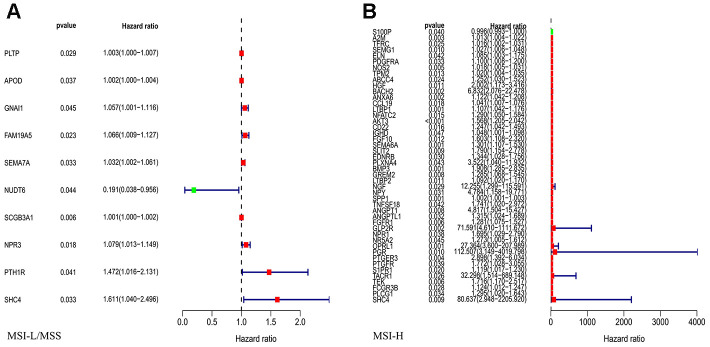
Univariate Cox regression analysis for the IRGs in MSI-L/MSS (**A**) or MSI-H (**B**) samples of gastric cancer to assess the prognostic value.

To assess the predictive value for OS, we performed univariate Cox regression analysis for all the differentially expressed IRGs between the MSI-L/MSS or MSI-H samples. Our analysis identified 10 and 46 genes as prognostic factors in MSI-L/MSS (Figure 2A) and MSI-H (Figure 2B) GC patients, respectively.

### Prognostic model construction in GC

The prognostic factors for OS identified by univariate Cox analysis were further analyzed in a multivariate Cox model. As a result, 6 and 46 genes were separately selected to further establish a predictive model in MSI-L/MSS patients or MSI-H patients. The prognostic risk score for MSI -L/MSS samples were determined using the formula below: Risk score = 0.028771494*(expression of *SEMA7A*) + (-2.12419407)*(expression of *NUDT6*) + 0.001719489*(expression of *SCGB3A1*) + 0.074754971*(expression of *NPR3*) + 0.348798938*(expression of *PTH1R*) + 0.501115829*(expression of *SHC4*). MSI-L/MSS patients were divided into low-risk and high-risk groups according to the median prognostic risk score. (Figure 3A) Patients’ survival status indicated that high-risk patients tended to have a worse prognosis than low-risk patients. (Figure 3A) The five prognostic genes (*SEMA7A, SCGB3A1, NPR3, PTH1R,* and *SHC4*) were more enriched in the high-risk group affirmed that they were all independent risk factors while the lower enriched gene (*NUDT6*) was a protective factor. (Figure 3A) The model was significantly associated with OS in MSI-L/MSS samples (p<0.01), with the higher survival rates in low-risk group than high-risk group. (Figure 3B) The AUC of the 1, 3, and 5-year survival were 0.624 (Figure 3C), 0.7 and 0.729. (Supplementary Figure 1A, 1B)

**Figure 3:**
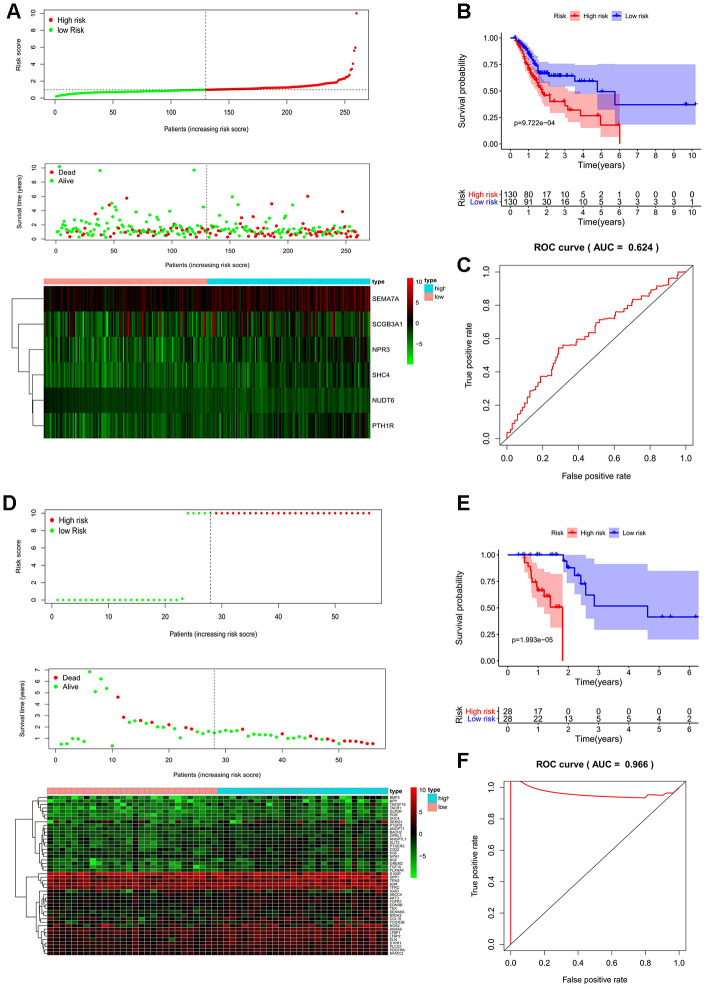
**Prognostic analyses used risk score model.** Candidate genes selected by univariate Cox regression were further analyzed in a multivariate Cox model for all MSI-L/MSS and MSI-H samples. The 17 prognostic genes of MSH patients determined by univariate Cox regression and Kaplan-Meier survival analysis were analyzed in a multivariate Cox model and 3 genes were acquired to construct a predictive model. (**A**, **D**, **G**) The distribution of risk score, survival status, and expression heat map. (**B**, **E**, **H**) The Kaplan-Meier curves for low-risk and high-risk groups. (**C**, **F**, **I**) The ROC curves for predicting OS by the risk score. AUC, area under the curve; ROC, receiver operator characteristic; OS, overall survival.

**Figure 3:**
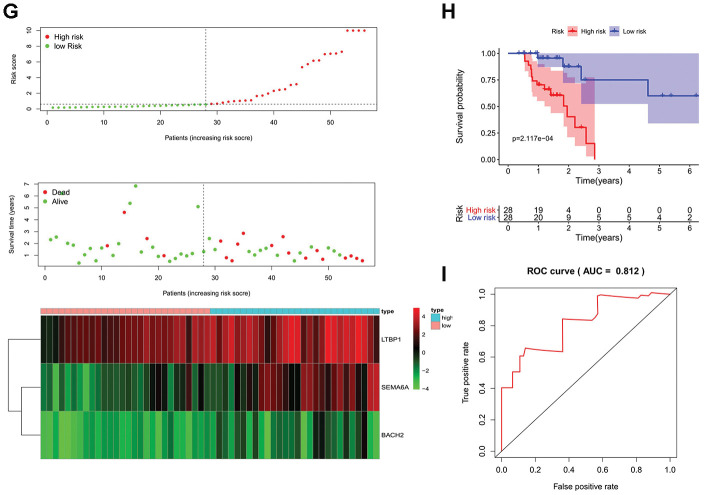
**Prognostic analyses used risk score model.** Candidate genes selected by univariate Cox regression were further analyzed in a multivariate Cox model for all MSI-L/MSS and MSI-H samples. The 17 prognostic genes of MSH patients determined by univariate Cox regression and Kaplan-Meier survival analysis were analyzed in a multivariate Cox model and 3 genes were acquired to construct a predictive model. (**A**, **D**, **G**) The distribution of risk score, survival status, and expression heat map. (**B**, **E**, **H**) The Kaplan-Meier curves for low-risk and high-risk groups. (**C**, **F**, **I**) The ROC curves for predicting OS by the risk score. AUC, area under the curve; ROC, receiver operator characteristic; OS, overall survival.

Next, a similar predictive model was established for patients with MSI-H status, (Figure 3D–3F). Furthermore, a newer reliable optimization model was established for increasing the stability of the model. Using this model, we observed that 17 of 46 prognostic genes analyzed by Kaplan-Meier survival were independent predictors of survival in GC patients with MSI-H (Supplementary Figure 2). These predictor genes were further analyzed using multivariate Cox regression model, and 3 genes were used to construct a predictive model based on the formula: Risk score = 0.363097478913475*(expression of SEMA6A) + 0.0923759499631374*(expression of LTBP1) + 1.84905247199979*(expression of BACH2). MSI-H patients were divided into low-risk and high-risk groups according to the median prognostic risk score (Figure 3G). And the survival analysis suggests that patients in high-risk group had an increased mortality risk (Figure 3G). The three prognostic genes were enriched in the high-risk group, suggesting that they were risk factors. (Figure 3G) The Kaplan-Meier curve showed higher survival rates in the low-risk group compared to the high-risk group. (Figure 3H) The AUC values of 1, 3, and 5 year survival of the model were 0.812, 0.891, and 0.832, respectively (Figure 3I, Supplementary Figure 1C, 1D), indicating that the predictive model had a high sensitivity and specificity.

### Validation of predictive model (risk score)

**Figure 4:**
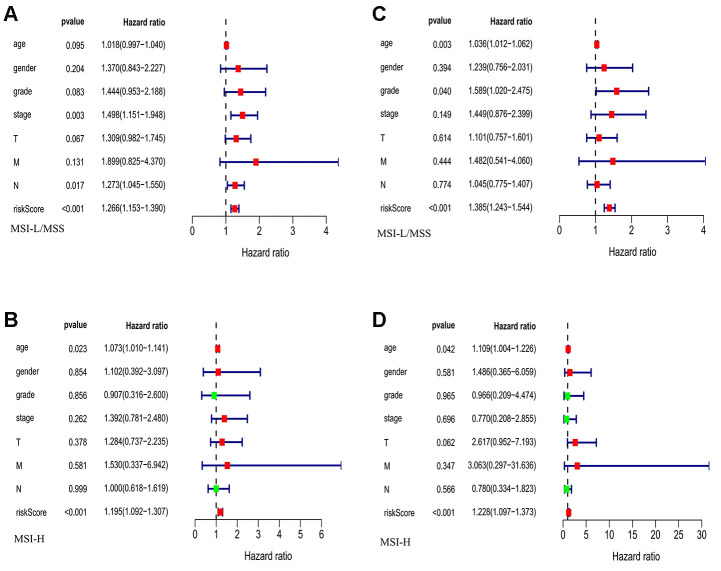
**The evaluation of the risk score and other clinicopathological factors.** (**A**, **B**) The Univariate regression analysis in MSI-L/MSS samples and MSI-H samples. (**C**, **D**) The multiple regression analysis in MSI-L/MSS samples and MSI-H samples.

Our univariate Cox regression analysis suggests a significant association of risk score with overall survival (OS) of GC patients in both MSI-L/MSS (*P*<0.001, Figure 4A) and MSI-H (*P*<0.001, Figure 4B). Furthermore, the multivariate regression Cox analysis suggests that risk score (Figure 4C), age and tumor grade are important factors that correlate with survival outcome in patients with MSI-L/MSS. Whereas, in case of MSI-H patients, only risk score (Figure 4D) and age serve as independent predictors of overall survival of the GC patients. And, both these analyses demonstrate the effective prognostic prediction by our model.

### Validation of immune cell infiltration for prognostic IRGs

**Figure 5:**
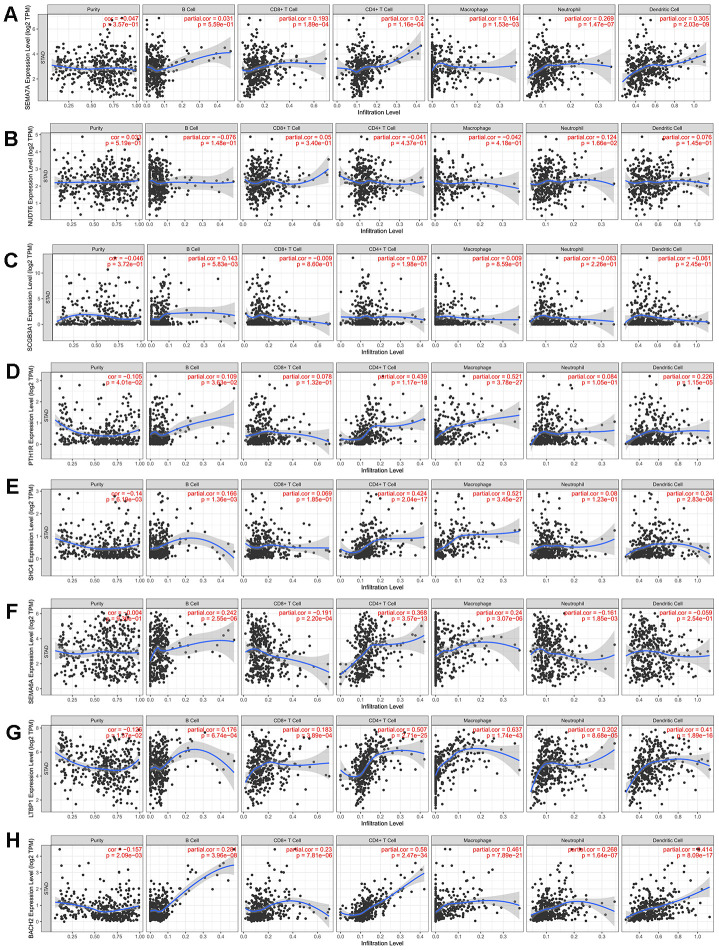
**Correlation between prognosis immune-related genes and immune cell infiltration.** (**A**–**E**) Expression levels of 5 prognosis immune-related genes for MSI-L patients have poor correlation with immune infiltration. (**F**–**H**) Three prognostic genes for MSI-H patients were significantly correlated with immune infiltration.

To verify the prognostic immune-related gene, we investigated the correlation between IRGs and immune cell infiltration via TIMER. As expected, factors in the two prognostic models had different immune infiltration conditions. The five prognostic genes of MSI-L patients have poor correlation with immune infiltration. In addition, there was good agreement between SCH4 and PTH1R which had no correlation with Neutrophil and CD8+ T Cell. (Table 1, Figure 5A–5E) However, three prognostic genes for MSI-H patients were significantly correlated with immune infiltration. (Table 1, Figure 5F–5H).

**Table 1:** Correlation of prognostic IRGs expression with immune infiltration level.

	**Gene**	**B Cell**		**CD8^+^ T Cell**		**CD4^+^ T Cell**		**Macrophage**		**Neutrophil**		**Dendritic Cell**	
		**Cor ^1^**	**p**	**Cor ^1^**	**p**	**Cor ^1^**	**p**	**Cor ^1^**	**p**	**Cor ^1^**	**p**	**Cor ^1^**	**p**
MSI-L	PTH1R	0.109	<0.05	0.078	0.132	0.439	<0.05	0.521	<0.05	0.084	0.105	0.226	<0.05
	SHC4	0.166	<0.05	0.069	0.185	0.424	<0.05	0.521	<0.05	0.080	0.123	0.240	<0.05
	NPR3	0.068	0.195	0.142	<0.05	0.271	<0.05	0.499	<0.05	0.140	<0.05	0.292	<0.05
	NUDT6	-0.076	0.148	0.050	0.340	-0.041	0.437	-0.042	0.418	0.124	<0.05	0.076	0.145
	SCGB3A1	0.143	<0.05	-0.009	0.860	0.067	0.198	0.009	0.859	-0.063	0.226	-0.061	0.245
	SEMA7A	0.031	0.559	0.193	<0.05	0.200	<0.05	0.164	<0.05	0.269	<0.05	0.305	<0.05
MSI-H	BACH2	0.281	<0.05	0.230	<0.05	0.580	<0.05	0.461	<0.05	0.268	<0.05	0.414	<0.05
	LTBP1	0.176	<0.05	0.183	<0.05	0.507	<0.05	0.637	<0.05	0.202	<0.05	0.410	<0.05
	SEMA6A	0.242	<0.05	-0.191	<0.05	0.368	<0.05	0.240	<0.05	-0.161	<0.05	-0.059	0.253

Abbreviations: ^1^ Correlation coefficient.

### TF regulatory network

**Figure 6:**
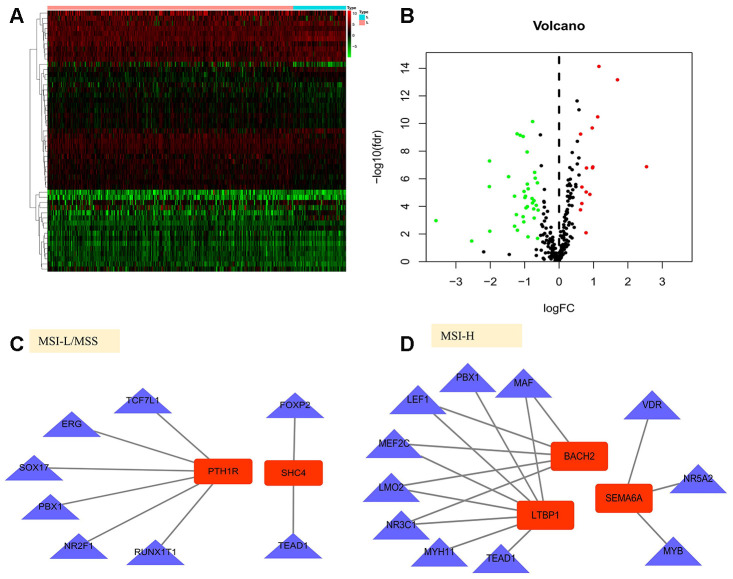
**Gene regulatory network in gastric cancer.** (**A**, **B**) Heatmap and volcano plot of the upregulated (red) and downregulated (green) transcription factors(TFs) between the MSI-L/MSS and MSI-H samples of gastric cancer. (**C**, **D**) TF-gene regulation networks in MSI-L/MSS or MSI-H samples. Red node stands for the hub gene and blue triangle stands for the transcription factor.

To investigate the potential molecular mechanisms of prognostic IRGs, we examined the expression profiles of transcription factors (TFs) and found that 51 were differentially expressed between MSI-H and MSI-L/MSS patients (Figure 6A, 6B). Further, we established the TF-gene regulatory network based on the correlation between TFs and prognostic genes (correlation score >0.4, *P*<0.01, Figure 6C, 6D).

### GSEA identified the prognostic IRGs-Related signaling pathway

To identify prognostic IRGs-associated signaling pathways in gastric cancer, we conducted Gene Set Enrichment Analysis (GSEA) between high and low prognostic IRGs expression data sets. GSEA indicated that these prognostic genes are strongly (FDR<0.25, *NP*<0.01) associated with different pathway, which accounted for the different oncogenic mechanisms. The top 5 GSEA terms are enlisted (Table 2).

**Table 2:** The top 5 GSEA terms of SHC4/PTH1R for MSI-L/MSS patients and LTBP1, SEMA6A, and BACH2 for MSI-H patients.

	**GENESET**	**ES**	**NES**	**NP**	**FDR**
BACH-2	PROTEASOME_PATHWAY	-0.9207	-2.1224	0	0.0034
	MCM_PATHWAY	-0.854	-1.9003	0.0039	0.0634
	FREE_PATHWAY	-0.8257	-1.864	0.0039	0.0656
	EDG1_PATHWAY	0.622	1.8559	0.006	0.2026
	ERYTH_PATHWAY	0.7518	1.8859	0.004	0.2208
	**GENESET**	**ES**	**NES**	**NP**	**FDR**
SEMA6A	AGPCR_PATHWAY	0.7132	1.8899	0	0.0506
	NO1_PATHWAY	0.6	1.9007	0.0041	0.0537
	DREAM_PATHWAY	0.6954	1.9493	0.0019	0.0386
	ACE2_PATHWAY	0.8377	1.9547	0	0.0548
	SHH_PATHWAY	0.7806	1.9935	0	0.0642
	**GENESET**	**ES**	**NES**	**NP**	**FDR**
LTBP1	PROTEASOME_PATHWAY	-0.9381	-2.2406	0	0.0008
	ACE2_PATHWAY	0.8829	2.0506	0	0.0087
	PLATELETAPP_PATHWAY	0.8336	2.0805	0	0.0084
	HDAC_PATHWAY	0.7302	2.1425	0	0.004
	NFAT_PATHWAY	0.6751	2.2128	0	0.0021
	**GENESET**	**ES**	**NES**	**NP**	**FDR**
PTH1R	EIF_PATHWAY	-0.8509	-2.0818	0	0.0131
	PROTEASOME_PATHWAY	-0.8889	-2.0779	0	0.0071
	CASPASE_PATHWAY	-0.8309	-2.0769	0	0.0049
	EIF2_PATHWAY	-0.8892	-2.0661	0	0.0046
	MITOCHONDRIA_PATHWAY	-0.7839	-2.0595	0	0.0039
	**GENESET**	**ES**	**NES**	**NP**	**FDR**
SHC4	PROTEASOME_PATHWAY	-0.9044	-2.1247	0	0.0057
	CASPASE_PATHWAY	-0.8097	-2.0801	0.002	0.0029
	MITOCHONDRIA_PATHWAY	-0.7654	-2.0214	0.0021	0.0054
	DNAFRAGMENT_PATHWAY	-0.9111	-2.011	0	0.0021
	SARS_PATHWAY	-0.8321	-1.9996	0	0.0042

### Clinical evaluation of SHC4/PTH1R for MSI-L/MSS patients

**Figure 7:**
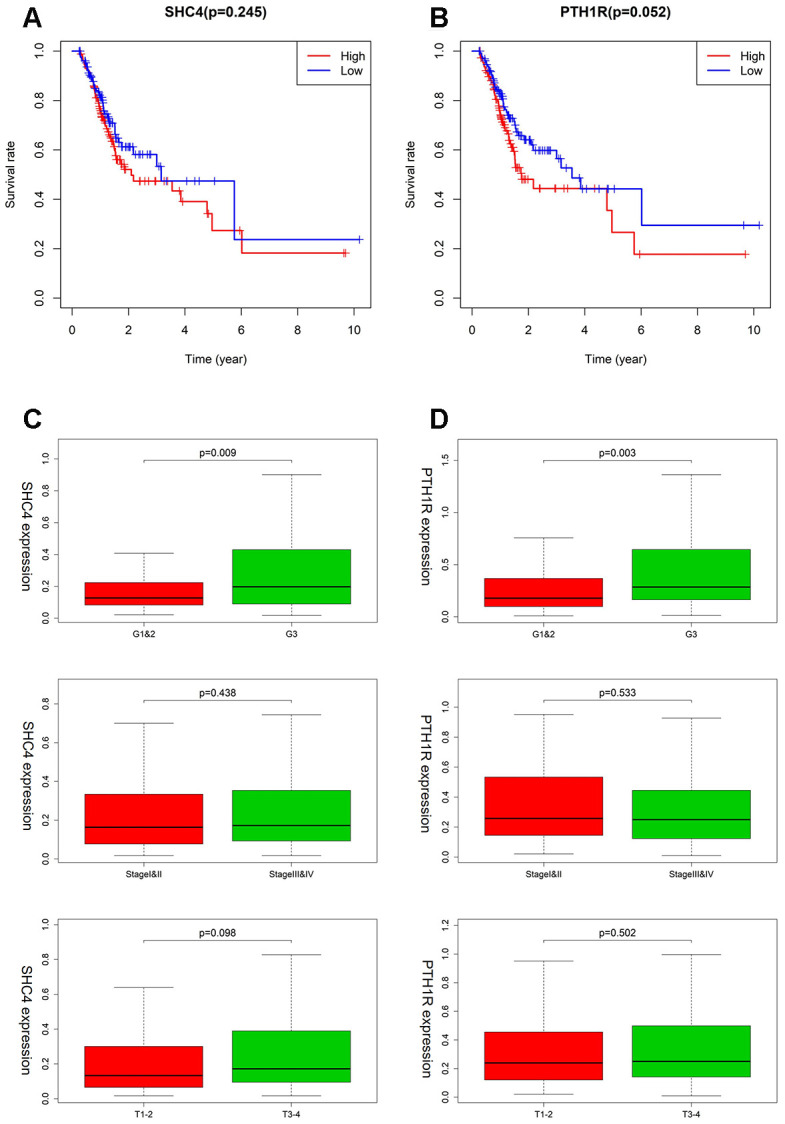
**The clinical evaluation of SHC4/PTH1R for MSI-L/MSS patients.** (**A**, **B**) Kaplan–Meier survival curves for SHC4/PTH1R in MSI-L/MSS patients. (**C**, **D**) Expression of SHC4/PTH1R in different grade stage, clinical stage, and T stage groups for MSI-L/MSS patients.

In the TF-IRG network, only SHC4 and PTH1R have connections with TF. In addition, for immune infiltration, there was good agreement between *SCH4* and *PTH1R* which had no correlation with Neutrophil and CD8+ T Cell. The survival analysis suggests that in case of MSI-L/MSS patients, *SHC4* and *PTH1R* can serve as risk factors (Figure 7A, 7B). Furthermore, patients with grade 3 (*P*=0.009) and T3+T4 stage (*P*=0.098) tumors showed increased expression levels of *SHC4* (Figure 7C). Whereas, *PTH1R* was highly expressed only in patients with grade 3 tumors (*P*=0.003, Figure 7D).

### Clinical utility of prognostic signature (LTBP1, SEMA6A, and BACH2) for MSI-H patients

**Figure 8:**
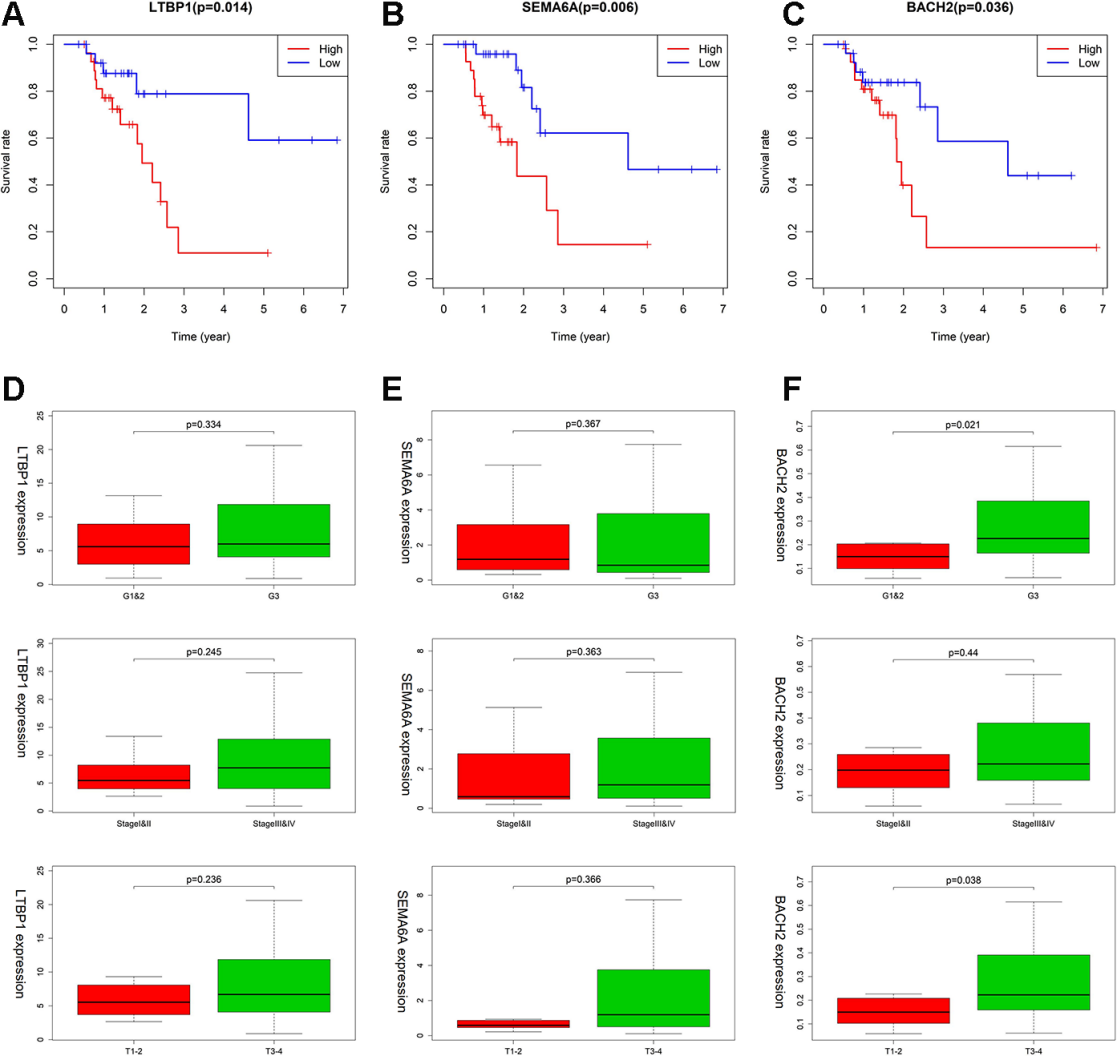
**The association between LTBP1, SEMA6A, and BACH2 expression and different clinical signatures in MSI-H samples.** (**A**–**C**) Kaplan–Meier survival curves for LTBP1, SEMA6A, and BACH2 in MSI-H samples. (**D**–**F**) Expression of LTBP1, SEMA6A, and BACH2 in different grade stage, clinical stage, and T stage groups.

As shown in Figure 8A, higher expression of LTBP1, SEMA6A, BACH2 was significantly related to worse OS (Figure 8A–8C). LTBP1 displayed an upregulated status in MSI-H patients with worsening tumor status, including the Grade 3 (*P* =0.334), stage III/IV (*P* =0.245), and T3 + T4 stage (*P* = 0.236) (Figure 8D). For SEMA6A, higher expression was observed in patients with stage III/IV (*P* =0.363) and T3 + T4 stage (*P* = 0.366), but it showed a lower expression in the Grade 3 group. (Figure 8E) *BACH2* showed an increased expression in GC patients with grade 3 (*P*=0.021) and T3+T4 stage (*P*=0.038) tumors (Figure 8F).

## DISCUSSION

Although surgical resection, chemotherapy, and targeted therapy are effective treatment methods, tumor recurrence and metastasis post treatment result in poor prognosis of gastric cancer [10–12]. Studies have shown that both tumor staging and heterogeneous molecular characterization could affect survival of GC patients [3, 13]. Currently, there are two comprehensive molecular subtypes of gastric cancer established by The Cancer Genome Atlas (TCGA) and the Asian Cancer Research Group (ACRG) [2, 13]. Of note, in either of the classifications, MSI-H was described as a significant subgroup with specific histopathological features and better prognosis [2, 3, 13].

As evident from a retrospective study, GC patients with MSI-H have a better prognosis only for stage II tumors [6]. The results of the MAGIC trial suggest that compared to patients with MSI-L/MSS, the ones with MSI-H status have better overall survival outcomes when treated with surgery alone as opposed to combined treatment with chemotherapy and surgery [14]. These results are consistent with other retrospective studies having GC patients harboring MSI-H stage II and III tumors [7, 15]. Interestingly, the MAGIC trial also indicates that the MSI-H tumors show abundant immune infiltration [14], suggesting that they could serve as better candidates to check the efficacy of the immune checkpoint blockade therapies [16, 17]. Perhaps because of the small sample size of this study, the efficacy of checkpoint inhibitors in MSI-H GC is not satisfactory [18, 19].

**Figure 9:**
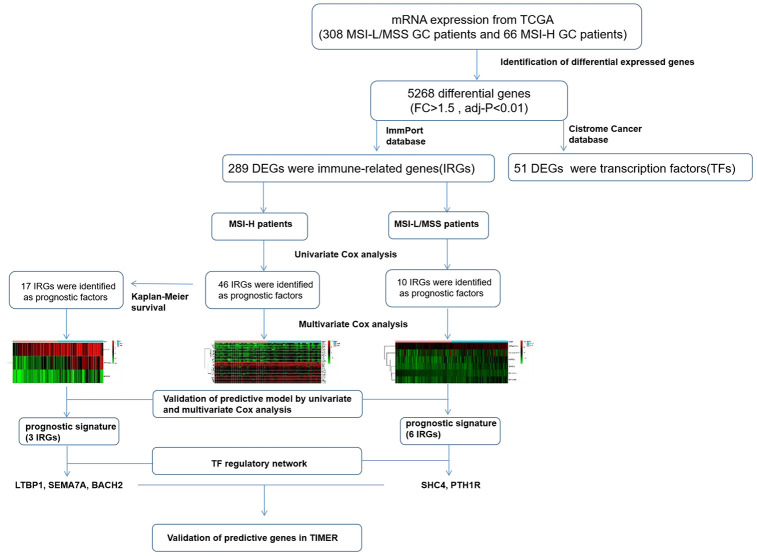
**The workflow of construction and validation of the immune signature.**

Although several studies discuss the prognostic significance of MSI-associated genes in GC [20], our study focused on identifying an immune-related gene expression signature where we have screened and analyzed all the DEGs between MSI-H and MSI-L/MSS patient samples to build a risk model and allow better prediction of patient prognosis. And the genes identified in MSI -L/MSS samples and MSI-H samples were totally different. Moreover, the unique signature patterns for each of these subtypes identified in our study predicted prognosis of patients with high accuracy. Furthermore, based on this model, we constructed a TF-IRG regulatory network to understand the underlying molecular mechanisms to help delineate their potential use in the clinics. Furthermore, GSEA indicated that these prognostic genes are strongly associated with different pathway, which accounted for the different oncogenic mechanisms. And our analysis provides distinct gene signatures for the MSI-H (*LTBP1, SEMA6A, BACH2*) and MSI-L/MSS (*SHC4, PTH1R*) patients, which could serve as potential candidates in the clinics. (Figure 9).

In case of the MSI-L/MSS subgroup, the significantly de-regulated gene *PTH1R* a member of the GPCR family of proteins, is defined as a type 1 parathyroid hormone receptor [21], which serves as an oncogene in breast cancer as silencing of *PTH1R* suppressed cell proliferation and induced apoptosis in breast cancer cells [22]. Furthermore, the co-expression of elevated levels of parathyroid hormone-related peptide (PTHrP) and PTH1R significantly correlated with cancer metastasis and mortality in patients with lung adenocarcinoma [23]. The results of our study are consistent with those reported by previous studies, where we observe that increased expression of the *PTH1R* correlated with an advanced tumor grade 3 and elevated mortality risk. However, these results are in contrast to another study with hepatocellular carcinoma where decreased expression of *PTH1R* was significantly associated with large tumor size and poor overall survival [24].

For MSI-H patients, our analysis indicated that the *BACH2* gene was significantly up-regulated and correlated with high grade tumor (Grade 3 and T3+T4) and poor survival outcome in patients. *BACH2* encodes a B-cell-specific transcription factor which plays an important role in regulating immune homeostasis and inflammatory responses. However, the role of BACH2 in tumorigenesis remains controversial and alters as per tissue and cancer type [25, 26, 27, 28]. For instance, BACH2 has been shown to function as tumor suppressor in mantle cell lymphoma (MCL) and diffuse large B-cell lymphoma (DLBCL), whereas in another study its expression has been associated with poor prognosis in DLBCL patients [29]. Consistent with this report, our study showed that higher expression of BACH2 was significantly related to worse OS. In addition, BACH2 promotes tumor growth through tumor immune suppression of Treg-mediated CD8+ T cells [30]. Therefore, studying the expression of *BACH2* may provide newer insights for the prognosis and design of immune-based therapies in MSI-H gastric cancer.

While our study was primarily based on the analysis of the RNA-seq expression data from publicly available datasets, the gene signature identified should possibly facilitate development of immunotherapeutic targets and improve the response rate of GC patients with MSI to immunotherapy. However, there remain several limitations in our study. First, we have relied heavily on the TCGA dataset, which provides less information about the treatment regimen applied for the patients. Second, the sample size in our study was small and this may have affected the efficacy and stability of the prediction model. Third, the prognostic genes identified in our study remain to be validated *in vitro* and *in vivo* using a larger cohort of actual patient samples.

## CONCLUSION

Our study provides a prognostic gene signature for predicting the OS of GC patients with MSI-H or MSI-L/MSS status. We have also explored the clinical utility of these prognostic IRGs by their statistically strong correlation with OS, tumor grad and clinical stage. We envision that the outcome of our study may help in understanding the underlying molecular mechanism, and interaction of immune microenvironment with tumor profile, which could allow patient-tailored treatment of GC patients.

## MATERIALS AND METHODS

### Data acquisition

Gene expression data and clinical information of GC patient samples identified with MSI were collected from the TCGA database (https://cancergenome.nih.gov/), which includes 66 MSI-H patients and 308 MSI-L/MSS patients (Table 3, until September 18, 2019). The expression data was HTSeq-FPKM type, by using Trimmed mean of M values (TMM) implemented in the edgeR Bioconductor package and filtering out genes whose expression was less than 0.2, the data was normalized. 2498 IRGs were accessed from the ImmPort database (https://www.immport.org/), whereas 317 transcription factors (TFs) were accessed from the Cistrome Cancer database (http://cistrome.org/).

**Table 3:** Clinical and pathological characteristics of GC patients.

**Size**	**Characteristics**	**Count 374**	**Percent (%) 100**
Sex			
	Male	241	64.40
	Female	133	35.50
Age(years)	65.80 ±10.65		
	≥60	251	67.11
	<60	123	32.89
Stage	I-II	163	43.58
	III-IV	188	50.27
	unknown	23	6.15
T-stage			
	T1-T2	99	26.50
	T3-T4	267	71.40
	Tx	8	2.10
Grade			
	G1 and G2	147	39.30
	G3	218	58.29
	Gx	9	2.41
MSI			
	MSI-H	66	17.65
	MSI-L/MSS	308	82.35

### Identification of differentially expressed genes

Basing on a log2 transformation of fold-change, differential genes (DGs) between MSI-H and MSI-L/MSS samples were screened by the R 3.5.2 software and edgeR package, with a cut-off criteria set as fold-change (FC) >1.5, and adjusted *P*-value (adj.*P*) <0.01. The differentially expressed IRGs and TFs were identified from differentially expressed mRNAs in the TCGA cohort using R software. The pheatmap package in R software was used to generate the heat-maps and volcano plots to allow stratification of GC patients based on their MSI status.

### Construction and validation of prognostic model

Univariate Cox regression analysis allowed the identification of 46 prognostic IRGs for 2498 genes of ImmPort database, which were then used to perform the Kaplan-Meier survival analysis in GC patients with MSI-H status, both using the Survival package of R software. Kaplan-Meier plots were generated to illustrate the relationship between patients’ overall survival and gene expression levels of IRGs which were stratified by the median. The relationship was tested by log-rank test, *P* values < 0.05 were considered to be statistically significant. These candidate genes were also analyzed using the multivariate Cox regression model to calculate the risk score (RS). GC patients with MSI-H or MSI-L/MSS status were distributed into either high-risk or low-risk disease groups based on their median prognostic RS. Furthermore, the pheatmap package (R software version 3.5.2) was used to distribute RS, survival status, and expression heat-maps for the high-risk and low-risk groups. The R 3.5.2 software also allowed analyses of the Kaplan-Meier survival curves (Survival R package) and the receiver-operating characteristic (ROC) curves (Survival ROC package). Prognostic independence was determined using univariate and multivariate Cox regression analysis of the RS and other clinic-pathological factors.

### Construction of TF regulatory network

The interactions between prognostic IRGs and TFs were confirmed using R software. Correlation score >0.4 and *P*<0.01 were used as the cut-off values. TF-gene regulatory network was remodeled using the Cytoscape software (version 3.7.1).

### The abundance of immune cells for prognostic IRGs

The abundance of six types of immune cells, ie, B cells, CD4^+^ T cells, CD8^+^ T cells, neutrophils, macrophages, and dendritic cells were determined using TIMER up to May 18, 2020 (https://cistrome.shinyapps.io/timer/). TIMER uses a previously reported statistical method to infer the abundance of tumor-infiltrating immune cells (TIICs) on the basis of gene expression profiles. A correlation between the expression levels of prognostic IRGs and the levels of TIICs were revealed via TIMER.

### Gene set enrichment analysis (GSEA)

GSEA was performed using GSEA 3.0 (http://www.broadinstitute.org/gsea/). A total of 374 gastric samples inthe TCGA cohort were divided into two groups accordingto the expression of prognostic IRGs (divided by median value). For each analysis, gene set permutations were performed 1000 times to obtain a normalized enrichment score (NES) and a enrichment score (ES). A normalized *P* (NP) < 0.01 and a false discovery rate (FDR) < 0.25 were set as the cutoff.

### Statistical analysis

All analyses were conducted using R software version 3.5.2. For all statistical tests, *P* values < 0.05 were considered to be statistically significant. The differentially expressed genes were confirmed by Wilcox test using R software. Associations between IRGs and OS were evaluated by Kaplan-Meier curves and univariate Cox Survival. Univariate and multivariate analyses were performed using Cox regression. AUC of the survival ROC curve was calculated by the survival ROC R software package. Wilcox test was performed to analyze correlations between prognostic IRGs and clinical features.

### Availability of data and materials

The datasets analyzed during the current study are publicly available from the following online databases: TCGA (https://tcga-data.nci.nih.gov/tcga/); ImmPort database (https://www.immport.org/); the Cistrome Cancer database (http://cistrome.org/); TIMER (https://cistrome.shinyapps.io/timer/).

### Ethics approval

The databases are publicly available and open to access, so this study did not need the approval from the ethics committee.

## Supplementary Material

Supplementary Figures
