# Supplementary material for: Immune-related gene signature predicts overall survival of gastric cancer patients with varying microsatellite instability status
Source: Aging (Albany NY). 2020 Dec 9;13(2):2418–35. doi: 10.18632/aging.202271 (PMC7880323; doi:10.18632/aging.202271)
Supplement: Supplementary Figures [file aging-13-202271-s001.pdf]

SUPPLEMENTARY FIGURES

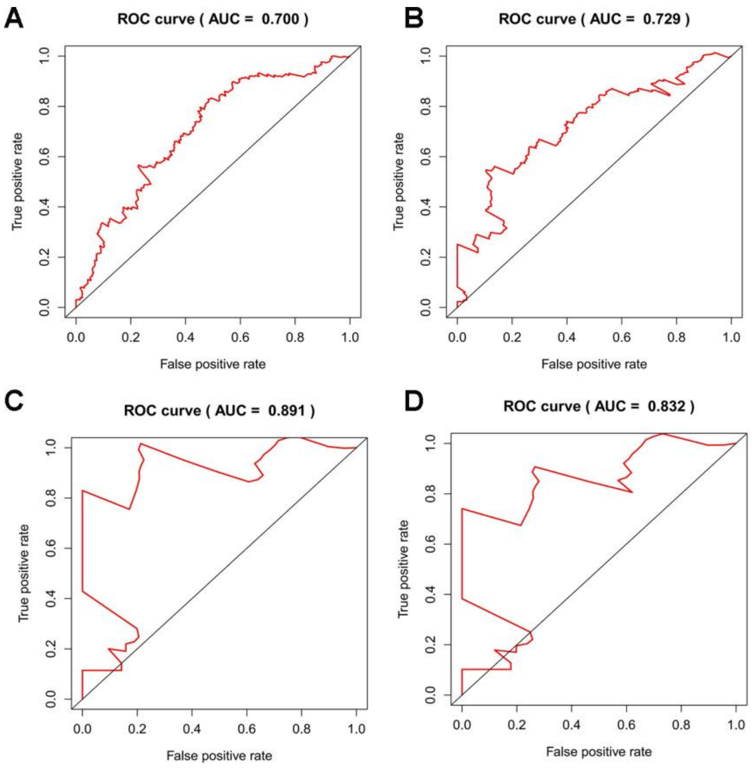

Supplementary Figure 1. The ROC curves for predicting OS by the risk score. (**A**, **B**) The AUC values of 3 and 5 year survival of the prognostic model in MSI-L/MSS patients. (**C**, **D**) The AUC values of 3 and 5 year survival of the prognostic model in MSI-H samples.

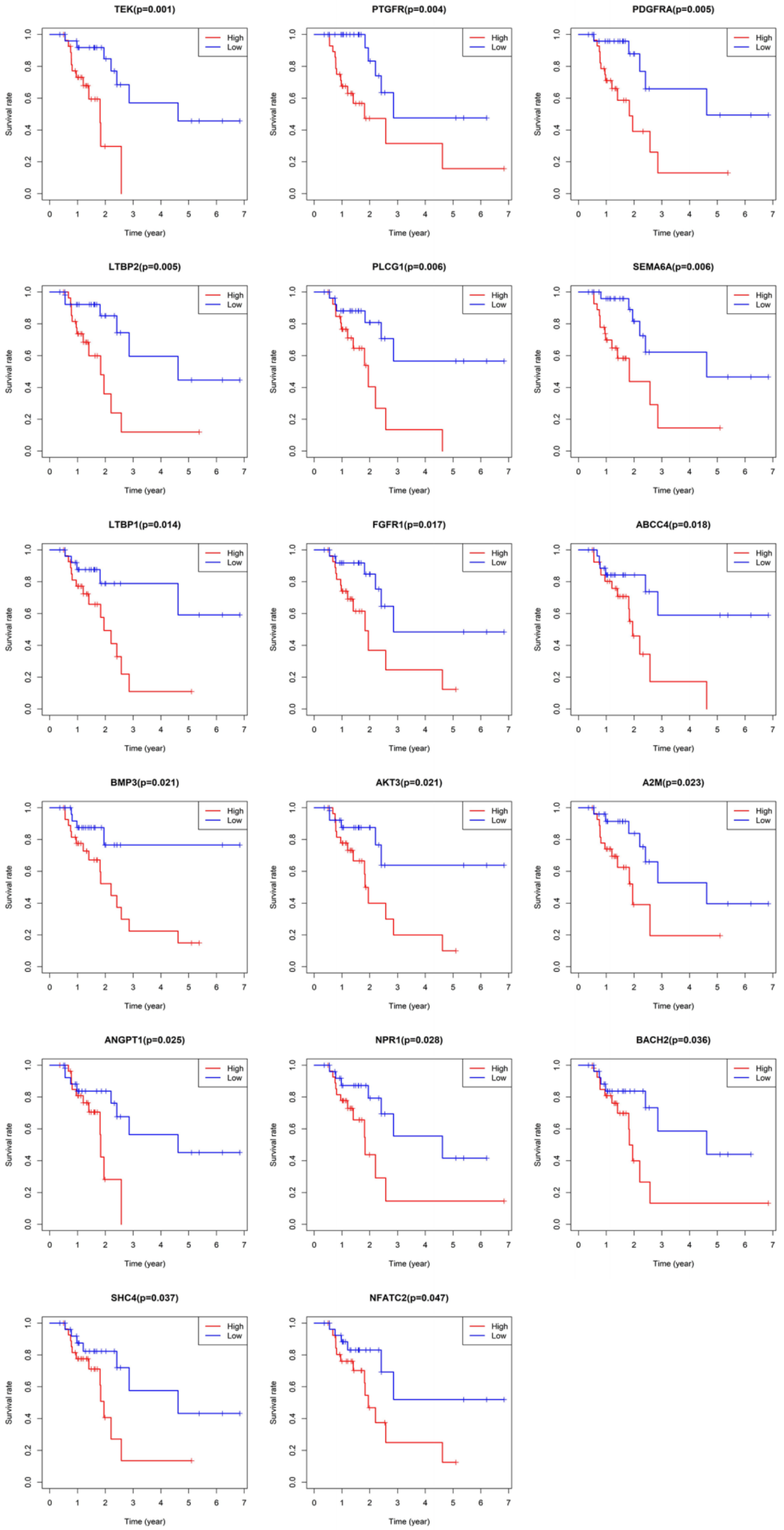

Supplementary Figure 2. Kaplan–Meier survival curves for identical immune genes associated with OS of MSI-H samples both in Kaplan-Meier survival and univariate regression.
